# Molecular cloning and characterization of the family of feline leucine-rich glioma-inactivated (LGI) genes, and mutational analysis in familial spontaneous epileptic cats

**DOI:** 10.1186/s12917-017-1308-9

**Published:** 2017-12-13

**Authors:** Yoshihiko Yu, Daisuke Hasegawa, Aki Fujiwara-Igarashi, Yuji Hamamoto, Shunta Mizoguchi, Takayuki Kuwabara, Michio Fujita

**Affiliations:** Department of Clinical Veterinary Medicine, Nippon Veterinary and Life Science University, 1-7-1 Kyonan-cho, Musashino-shi, Tokyo, 180-8602 Japan

**Keywords:** Feline, Epilepsy, Lgi, Cloning and sequence analysis, Molecular characterization

## Abstract

**Background:**

Leucine-rich glioma-inactivated (LGI) proteins play a critical role in synaptic transmission. Dysfunction of these genes and encoded proteins is associated with neurological disorders such as genetic epilepsy or autoimmune limbic encephalitis in animals and human. Familial spontaneous epileptic cats (FSECs) are the only feline strain and animal model of familial temporal lobe epilepsy. The seizure semiology of FSECs comprises recurrent limbic seizures with or without evolution into generalized epileptic seizures, while cats with antibodies against voltage-gated potassium channel complexed/LGI1 show limbic encephalitis and recurrent limbic seizures. However, it remains unclear whether the genetics underlying FSECs are associated with LGI family genes. In the present study, we cloned and characterized the feline LGI1–4 genes and examined their association with FSECs. Conventional PCR techniques were performed for cloning and mutational analysis. Characterization was predicted using bioinformatics software.

**Results:**

The cDNAs of feline *LGI1–4* contained 1674-bp, 1650-bp, 1647-bp, and 1617-bp open reading frames, respectively, and encoded proteins comprising 557, 549, 548, and 538 amino acid residues, respectively. The feline LGI1–4 putative protein sequences showed high homology with *Homo sapiens*, *Canis familiaris*, *Bos taurus*, *Sus scrofa*, and *Equus caballus* (92%–100%). Mutational analysis in 8 FSECs and 8 controls for LGI family genes revealed 3 non-synonymous and 14 synonymous single nucleotide polymorphisms in the coding region. Only one non-synonymous single nucleotide polymorphism in LGI4 was found in 3 out of 8 FSECs. Using three separate computational tools, this mutation was not predicted to be disease causing. No co-segregation of the disease was found with any variant.

**Conclusions:**

We cloned the cDNAs of the four feline LGI genes, analyzed the amino acid sequences, and revealed that epilepsy in FSEC is not a monogenic disorder associated with LGI genes.

**Electronic supplementary material:**

The online version of this article (10.1186/s12917-017-1308-9) contains supplementary material, which is available to authorized users.

## Background

Epilepsy is a chronic functional neurological disorder characterized by recurrent, unprovoked epileptic seizures and a range of loss of consciousness, salivation, muscle spasms, and severe and prolonged convulsion. Epilepsy affects over 50 million people worldwide, and is one of the most common neurological diseases [1]. Although the causes of epilepsy are variable, approximately 70% is believed to be associated with genetic factors [2]. Thus, understanding the genetic causes is important for developing effective therapeutic strategies for epilepsy [3–5]. Recently, it was also reported that treatment with a chemical corrector, 4-phenylbutyrate ameliorated the increased seizure susceptibility of the LGI1E383A model mouse, a mouse model of familial epilepsy [6].

In 2009, we identified a feline family line with spontaneous recurrent epileptic seizures, and established a colony strain of spontaneous epileptic cats (familial spontaneous epileptic cats [FSECs]) [7]. The FSEC colony consists of large multigenerational pedigrees with multiple cases. FSECs are healthy except for the presence of recurrent epileptic seizures, which have a variable frequency between FSECs. FSECs have two types of clinical seizures, with some FSECs showing both or one seizure pattern(s), while others are asymptomatic. Spontaneous focal limbic seizures with or without evolving into generalized epileptic seizures is one type of seizure observed in FSECs. This seizure type is similar to the historical limbic kindling and/or kainate model of epilepsy [8], as also represents the common seizure type in cats [9–11]. Vestibular stimulation-induced generalized epileptic seizures is the other seizure type in FSECs, and is similar to the widely used EL mouse model, which shows genetic epilepsy [12]. As both the kindling/kainate model and the EL mouse are used to model human mesial temporal lobe epilepsy (MTLE), we consider that FSECs are a natural genetic model of human and feline TLE. We previously reported their clinical, electrophysiological and magnetic resonance imaging findings, which resembled those of human MTLE [13–16]. Further, a familial form of MTLE (FMTLE) was reported in humans [17–20]. Thus, our studies provide support that the FSEC is an animal model of human FMTLE. As such, understanding the genetic causes of FSECs may be useful in exploring the genetics of familial epilepsy.

The leucine-rich glioma-inactivated (LGI) protein family (LGI1–4) play important roles in the development and function of the vertebrate nervous system, including synaptic transmission and myelination. Indeed, dysfunction of these genes and encoded proteins are associated with neurological disorders including genetic epilepsy or autoimmune limbic encephalitis in animals and human [21, 22]. LGI1 has been shown to be causative in the lateral form of familial TLE (also termed autosomal dominant lateral TLE) [23–25]. In veterinary medicine, feline complex partial seizures with orofacial involvement (FEPSO; also termed feline TLE), characterized by recurrent limbic seizures (e.g., episodic orofacial automatism) with salivation, chewing, licking, and facial twitching, was previously reported [26–28]. Some of those cats have increased antibodies against the voltage-gated potassium channel (VGKC)/LGI1 complex [27]. Thus, FEPSO is considered to involve, at least in part, VGKC/LGI1 complex antibody-mediated limbic encephalitis (LE), resembling LE in humans.

Interestingly, while FSEC was associated with loss of neurons in the CA3 subarea of the hippocampus [13, 29], the VGKC/LGI1 complex antibody LE in humans is associated with focal CA3 atrophy [30]. A protein-truncating nonsense mutation in the LGI2 gene was identified as a cause of benign familial juvenile epilepsy in the Lagotto Romagnolo dog [31]. Further, LGI4 may contribute to susceptibility to benign familial infantile convulsions and childhood absence epilepsy in humans [32, 33]. Despite these overall findings, there has been no known association of the LGI3 gene with human or veterinary diseases, although LGI3 is highly expressed in the mouse brain in a developmentally- and transcriptionally-regulated manner [34]. As LGI gene family members are highly conserved and have the same overall structure, they may also have similar functions [21], including a predisposition to epilepsy.

In the present study, we examined the hypothesis that the etiology of FSECs is associated with LGI protein family dysfunction [22]. As the cDNA sequences of the feline LGI (fLGI) genes were not previously reported, we first cloned the cDNA of LGI family genes in the *Felis catus*, predicted the corresponding protein sequence, obtained their basic physical and chemical properties, and then performed phylogenetic and structural analysis. Next, we performed fLGI mutational analysis in FSECs and healthy cats to determine their roles in epilepsy.

## Results

### Characterization of full-length feline LGI cDNAs

We cloned and sequenced the fLGI1, fLGI2, fLGI3, and fLGI4 cDNAs, and sequences were submitted to the DNA Databank of Japan under accession numbers LC309277, LC309278, LC309279, and LC309280, respectively. Nucleotide sequence analysis showed that fLGI1 cDNA contained a 1674-base pair (bp) open reading frame (ORF) encoding a 557-amino acid peptide, fLGI2 cDNA contained a 1650-bp ORF encoding a 549-amino acid peptide, fLGI3 cDNA contained a 1647-bp ORF encoding a 548-amino acid peptide, and fLGI4 cDNA contained a 1617-bp ORF encoding a 538-amino acid peptide.

### Amino acid sequence analysis of the feline LGI proteins

**Fig. 1:**
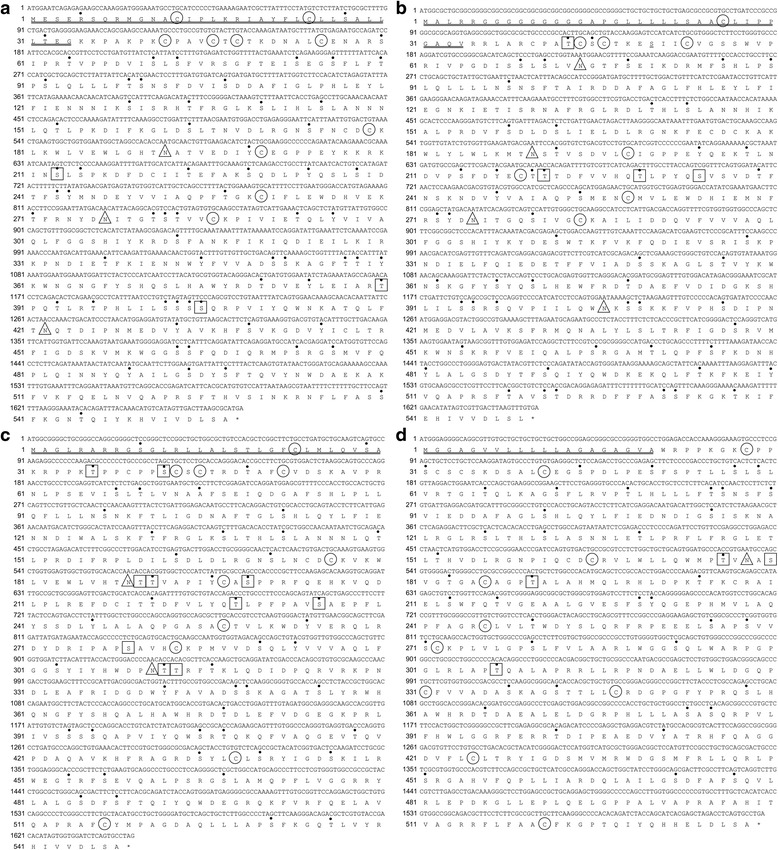
Nucleotide and deduced amino acid sequences of feline leucine-rich glioma-inactivated (fLGI) genes. **a** LGI1, **b** fLGI2, **c** fLGI3, and **d** fLGI4. Residues are numbered from the 5′ end of the open reading frame (ORF). The putative amino acid sequences are shown by the single-letter amino acid codes. Asterisk indicates the stop codon. The signal peptide sequence is double-underlined. The cysteine residues required for formation of disulfide bonds are indicated with circles. The putative N-linked or O-linked glycosylation sites are indicated with triangles or squares, respectively. The predicted phosphorylation sites are indicated by a dot above the single-letter amino acid code. The sequences reported in this paper have been deposited in the DNA Data Bank of Japan (DDBJ) (LC309277, LC309278, LC309279, and LC309280)

The physicochemical properties of the four LGI proteins were predicted using ProtParam software (Table 1). The fLGI1, fLGI2, fLGI3, and fLGI4 proteins were predicted to have a signal peptide located at 1–34, 1–34, 1–30, and 1–20 amino acids, respectively; three, four, ten, and four O-glycosylation sites, respectively; three, four, three, and one N-glycosylation sites, respectively; 66, 57, 50, and 44 phosphorylation sites, respectively; and five, four, five, and five disulfide bonds, respectively (Fig. 1). The subcellular distribution of the fLGI protein was predicted and summarized in Additional file 1.

**Table 1:** Physiochemical characteristics of the feline leucine-rich glioma-inactivated (LGI) proteins

Physiochemical characteristics	LGI1	LGI2	LGI3	LGI4
Amino acid	557	549	548	538
Total number of atoms	8959	8728	8683	8323
Molecular weight (Da)	63,817.99	62,249.12	61,887.77	58,979.10
Isoelectric point	8.39	6.21	8.46	7.60
Asp + Glu	56	63	51	49
Arg + Lys	61	58	56	50
Extinction coefficients	84520^a^/83770^b^	80955^a^/80330^b^	91050^a^/90300^b^	71805^a^/70930^b^
Aliphatic index	85.75	88.23	88.47	97.25
Instability index	42.89	53.34	44.27	47.50
Grand average of hydropathicity	−0.227	−0.119	−0.143	0.064

^a^Computed value based on the assumption that all cysteine residues appear as half cysteine’s; ^b^Assuming that no cysteine appears as a half cysteine

### Prediction of the structures and features of feline LGI proteins

**Fig. 2:**
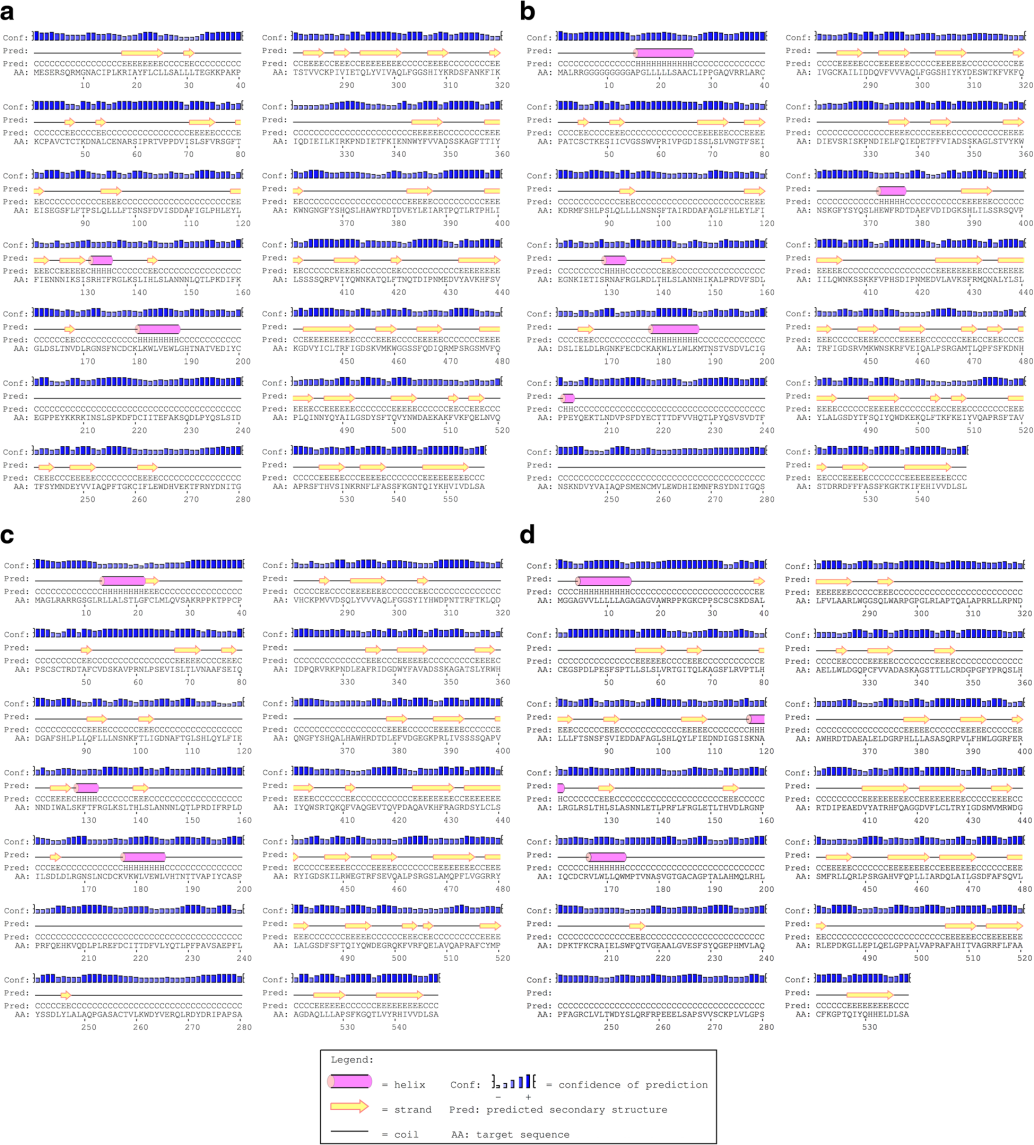
The predicted secondary structure of the fLGI proteins. Secondary structure of (**a**) fLGI1, (**b**) fLGI2, (**c**) fLGI3, and (**d**) fLGI4

**Fig. 3:**
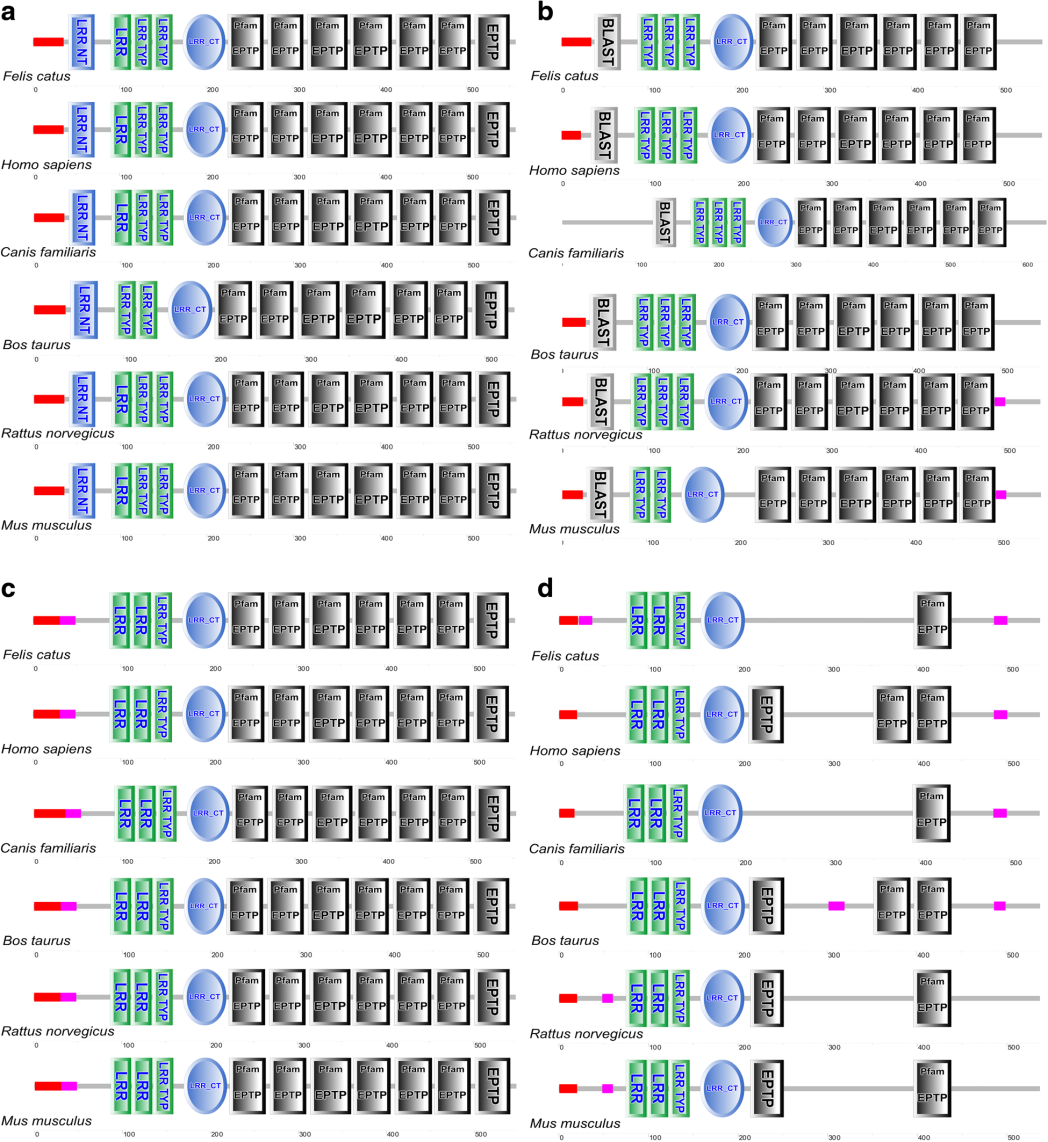
Confidently predicted domains, repeats, motifs, and features using SMART (Simple Modular Architecture Research Tool). This analysis included *Felis catus*, *Homo sapiens*, *Canis familiaris*, *Bos taurus*, *Rattus norvegicus*, and *Mus musculus*. LRR NT: Leucine rich repeat N-terminal domain; LRR: Leucine-rich repeats; LRR TYP: Leucine-rich repeats, typical subfamily; LRR_CT: Leucine rich repeat C-terminal domain; EPTP: Epitempin repeat. Note that BLAST also shows LRR NT. **a** LGI1, **b** LGI2, **c** LGI3, and **d** LGI4

Secondary structural analysis predicted the proportion of alpha helix, beta sheet, and random coils of the putative fLGI proteins as: fLGI1: 2.15%, 30.70%, and 7.15%, respectively; fLGI2: 5.65%, 24.95%, and 69.40%, respectively; fLGI3: 3.65%, 25.73%, and 70.62%, respectively; and fLGI4: 3.90%, 24.90%, and 71.20%, respectively (Fig. 2). Analysis of each deduced amino acid sequence using SMART (Simple Modular Architecture Research Tool) revealed that the leucine-rich repeat domain flanked by the cysteine-rich region (C-terminal) at the N-terminal followed by epitempin (EPTP) domain(s) was common to all fLGI proteins (Fig. 3, Additional file 2). While SMART showed two or three EPTP domains within the LGI4 protein in other mammals, with the exception of *Felis catus* and *Canis familiaris* (see Fig. 3), two EPTP domains of fLGI4 positioned in the corresponding regions to other mammals were excluded from the diagram because of E-value thresholds of 0.00075 and 0.0039. Further, the fLGI family proteins were predicted not to have an interaction in their respective pathways (Additional file 3).

### Exonic structure analysis, multiple alignments, and phylogenetic analysis

The fLGI1–3 genes included eight exons and seven introns, while the fLGI4 gene included nine exons and eight introns. All four genes shared a similar exon structure with those of other mammals (Additional file 4) based on the amino acid and genomic sequences listed in Additional file 5. Homology analysis indicated that the amino acid sequences of the predicted fLGI1–4 proteins were 94.7, 92.7, 88.5, and 86.3% identical, respectively, to the other species listed in Table 2. However, when compared with most typical mammalians including *Homo sapiens*, *Canis familiaris*, *Bos taurus*, *Sus scrofa* and *Equus caballus*, the identities were over 92%.

**Table 2:** Identity of amino acid sequences of the LGI family in other species

Species	LGI1 (%)	LGI2 (%)	LGI3 (%)	LGI4 (%)
*Homo sapiens*	99.3	96.2	97.6	93.5
*Rattus norvegicus*	97.1	95.1	96.5	88.1
*Mus musculus*	97.1	85.7	97.1	89.0
*Canis familiaris*	100.0	96.8	96.9	96.1
*Bos taurus*	95.0	96.2	96.7	92.9
*Sus scrofa*	99.1	96.6	98.4	92.8
*Equus caballus*	99.5	97.5	98.0	92.9
*Gallus gallus*	84.5	87.0	73.9	N/A
*Xenopus tropicalis*	80.7	81.6	N/A	43.7
All of the above species	94.7	92.7	88.5	86.3

*N/A* Not available

**Fig. 4:**
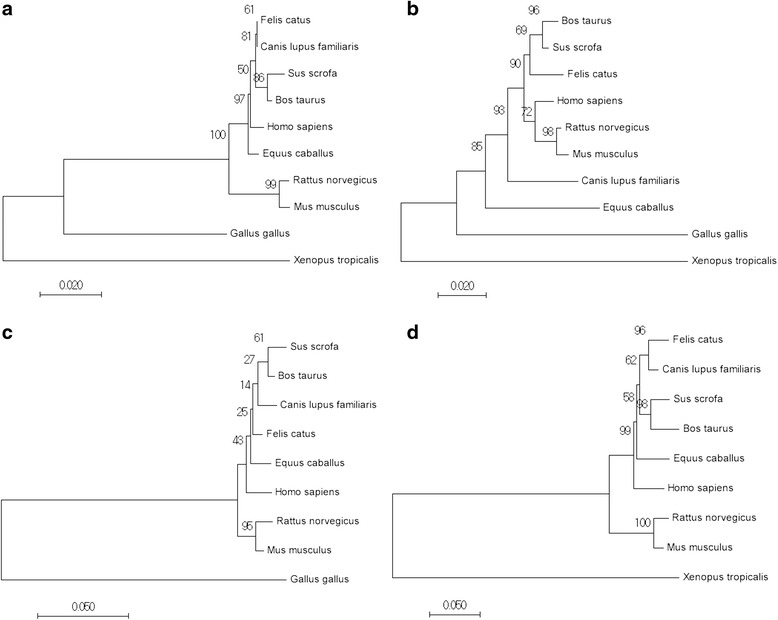
Phylogenetic trees of the LGI family. The phylogenetic trees of the LGI family proteins were constructed to investigate the evolutionary relationships using the neighbor-joining method with Molecular Evolutionary Genetics Analysis Version (MEGA) 7.0 to calculate the p-distance, 1000 bootstrap replication, and pairwise deletion for the gaps/missing data treatment. Amino acid sequences of the LGI family used for this construction were obtained from GenBank, and their accession numbers are shown in Additional file 10. **a** LGI1 protein, **b** LGI2 protein, **c** LGI3 protein, and **d** LGI4 protein. The numbers at the nodes indicate bootstrap majority consensus values from 1000 replicates

The amino acid sequences of the LGI proteins were aligned with sequences from other species using DNAMAN version 9.122 (Trial version; Lynnon Biosoft, San Ramon, CA, USA) (Additional files 6, 7, 8 and 9). Phylogenetic trees were constructed using MEGA 7.0 (Molecular Evolutionary Genetics Analysis Version 7.0) based on the amino acid sequences of the LGI family listed in Additional file 10 (Fig. 4), and included the proteins identified as homologous to LGI 1–4. The evolutionary history was inferred using the neighbor-joining method. The optimal trees with branch length sums of 0.27374760, 0.40249836, 0.38710421, and 0.76533180 are shown. The trees are drawn to scale, with branch lengths in the same units as those of the evolutionary distances used to infer the phylogenetic tree. While phylogenetic tree analysis based on the available amino acid sequences revealed that fLGI1, fLGI3, and fLGI4 shared the closest evolutionary relationship with *Canis familiaris*, fLGI2 showed the closest evolutionary relationship with *Sus scrofa* and *Bos taurus*.

### Mutational analysis and allele frequency

Except for exon1 of fLGI2, all exons that contained coding sequences were amplified from the genomic DNA from eight FSECs and eight control cats. It was impossible to design primers for exon 1 of fLGI2 because of its GC rich content. PCR products were compared with feline reference genomic sequences (NC_018733.2, NC_018726.2, NC_018726.2, and NC_018737.2). Sequence analysis of the fLGI gene family revealed 41 variants, the majority of which were not in the National Center for Biotechnology Information single nucleotide polymorphism (SNP) database (https://www.ncbi.nlm.nih.gov/projects/SNP/). Among them, three non-synonymous and 14 synonymous SNPs in the coding region were found. The three SNPs were found in *fLGI1*, which included one synonymous SNPs in the coding region and two SNPs in the intron region. Six synonymous SNPs in the coding region and 11 SNPs in the intron and non-coding regions were found in *fLGI2*. As for *fLGI3*, one non-synonymous SNP, four synonymous SNPs, and four intronic SNPs were detected. In fLGI4, two non-synonymous SNPs, three synonymous SNPs, and seven intronic SNPs were found. For the three non-synonymous SNPs found in the *fLGI* genes, P33A in *fLGI3* was heterozygous in a control cat, A17T in *fLGI4* was also heterozygous in a control cat, while E49K in *fLGI4* was heterozygous in three FSECs. No co-segregation of the disease was found with any variant. These results are summarized in Table 3 and Additional file 11.

**Table 3:** Allelic and genotypic distribution of non-synonymous mutation found in LGI1–4 in familial spontaneous epileptic cats (FSECs) and controls

	Nucleotide	Amino Acid	SIFT prediction	PP2 prediction	PROVEAN prediction	Group (n)	Genotype frequencies			Allele frequencies	
LGI3							C/C	C/G	G/G	f(C)	f(G)
	c.97C > G	p.P33A	Deleteriouss	Benign	Neutral	FSECs(8)	8	0	0	1	0
						Controls(8)	7	1	0	0.9375	0.0625
LGI4							G/G	G/A	A/A	f(G)	f(A)
	c.49G > A	p.A17T	Tolerated	Unknown	Neutral	FSECs(8)	8	0	0	1	0
						Controls(8)	7	1	0	0.9375	0.0625
							G/G	G/A	A/A	f(G)	f(A)
	c.145G > A	p.E49K	Tolerated	Benign	Neutral	FSECs(8)	5	3	0	0.8125	0.1875
						Controls(8)	8	0	0	1	0

In silico functional analysis for one non-synonymous SNP (P33A) in *fLGI3* and two non-synonymous SNPs (A17T, E49K) in *fLGI4* were performed using SIFT (Sorting Intolerant From Tolerant), PolyPhen-2 (Polymorphism Phenotyping v2), and PROVEAN (Protein Variation Effect Analyzer). SIFT predicted P33A in *fLGI3* as deleterious, and A17T and E49K in *fLGI4* as tolerated (SIFT scores were 0.02, 0.26, and 0.27, respectively). PP2 predicted P33A in *fLGI3* as benign, A17T in *fLGI4* as unknown, and E49K in *fLGI4* as benign (PP2 scores were 0.002, not available, and 0.185, respectively). PROVEAN predicted all SNPs as neutral (PROVEAN scores were −0.528, −0.346, and 0.948, respectively). These results are also summarized in Table 3.

## Discussion

LGI proteins are known to play an important role in synaptic transmission, and their dysfunction may cause neuronal hyperexcitability [22]. Although LGI1 gene mutations can cause autosomal dominant lateral TLE in humans (but not FMTLE), *LGI1* conditional knockout mice were reported to have epileptic discharges that started in the hippocampus [35]. Similarly, we previously reported that epileptic seizures in FSECs originated from the hippocampus and/or amygdala [13]. Thus, these data suggest that *LGI1* dysfunction may cause seizure onset in the mesial temporal lobe. Mutations of the LGI gene family are also associated with genetic epilepsy in both humans and animals [23–25, 31–33], although the association of *LGI3* with disease occurrence remains unclear. Nevertheless, LGI3 was reported to be broadly expressed in the adult mouse brain [34], and to play a regulatory role in neuronal exocytosis [36]. In the present study, we cloned cDNA of the fLGI gene family from feline brain tissue, analyzed their potential functions, and assessed their associations with feline familial epilepsy.

All fLGI family members cloned in this study showed a predicted molecular mass of approximately 60 kDa, similar to those in humans [37]. All fLGI family genes were predicted to have a signal peptide followed by a leucine-rich repeat domain flanked by cysteine-rich sequences, consistent with the reported previously features in humans [38]. Exonic structural analysis indicated conserved exonic numbers and bps, especially between mammalian species. Further, multiple alignments, homology analysis, and phylogenic tree analysis revealed that the LGI amino acid sequences were conserved with sequences of their analogous in other species, particularly in *Homo sapiens*, *Canis familiaris*, *Bos taurus*, *Sus scrofa*, and *Equus caballus* (>90% homology). LGI1 showed the highest conserved amino acid sequence, suggesting that it may have similar functions to those in other species. Domain analysis also showed conservative structures between mammalian species. Although there were occasional mismatched structures in some mammalian sequences, those sequences were considered not completely accurate. Interaction analysis suggested that the LGI1, 3, and 4 proteins interacted with the a disintegrin and metalloprotease (ADAM) family proteins. ADAM22 and ADAM23 genes were reported to be associated with progressive encephalopathy with epilepsy in human, and with genetic epilepsy in canine, respectively [39–41]. Thus, LGI-ADAM interactions are an area of important focus for future epilepsy research.

In our mutational analysis, we only evaluated exons and exon-intron boundaries, except for exon1 of *fLGI2* (we were unable to design the primers because of the high GC content). Thus, we cannot exclude the possibility that haplotypes within genomic regions of fLGI family genes may be associated with FSEC. Additionally, we cannot exclude other types of intronic mutations that could also cause splicing disruptions. Therefore, there is still a possibility that fLGI genes may be causative of epilepsy in FSECs. Furthermore, c.145G > A in *fLGI4* which caused E49K was seen in the three affected cats, suggesting this mutation could potentially cause increased risk for epilepsy. We found that FSECs did not have any coding mutations concordant for causing epilepsy in these genes. In veterinary medicine, VGKC/LGI1 complex antibody-mediated LE in feline TLE [27], and the *LGI2* mutation in benign familial juvenile epilepsy in Lagotto Romagnolo dogs [31], are the only two known diseases associated with LGI genes/proteins. Thus, further studies are required to determine the association of LGI proteins and genes with animal disease. It was reported that rats with a *LGI1* missense mutation (L385R) has sound-induced generalized tonic-clonic seizures [42].

Several forms of human familial TLE have been reported, including autosomal dominant and complex inheritance [17–20]. The initial pedigree analysis at the time we identified FSEC, presumed that FSEC might have an autosomal recessive inheritance pattern [7], however, the current extended pedigree also implied the possibilities for other inheritance patterns, such as low-penetrance autosomal dominant or complex inheritance as well. Genetic studies have been performed on multiple human families with FMTLE, and several loci have been mapped in large pedigrees by linkage analysis [43–47]. Most of these loci have not been replicated in other families, potentially because of their genetic heterogeneity. Only one of these studies performed whole exome sequencing in addition to linkage analysis, although no putative pathogenic variants were found in the region suggested by linkage analysis [47]. It is possible that whole genome sequencing combined with linkage analysis may reveal the genetic cause of human FMTLE, such as regulatory variants or risk haplotype outside of the coding regions, or genomic rearrangement. Recently, genome-wide analyses including linkage analysis, the transmission disequilibrium test, genome-wide association analysis using the feline SNP array, and whole genome sequencing have become available for feline genetic disorders [48, 49], providing new possibilities for genetic analysis of FSECs.

## Conclusion

In the present study, we cloned, sequenced, and characterized the cDNA sequences of the feline LGI1–4 genes. Using deduced amino acid sequences, we also predicted their physical and chemical properties, performed structural analyses, evaluated the evolutionary association between species, and examined their association with disease in FSECs. We found that the fLGI1–4 amino acid sequences were highly conserved with those of other mammals. However, mutational analysis revealed that the non-synonymous substitutions found in the fLGI family genes were not involved in disease occurrence in FSECs. Further studies using genome-wide strategies, including combinations of linkage analysis, the transmission disequilibrium test, or next generation sequencing, are required to identify the genes involved in epilepsy in FSECs. Nevertheless, our findings provide a further understanding of the functions of the fLGI genes and proteins, and emphasize the presence of FMTLE unrelated to only single mutations within coding regions of LGI family genes.

## Methods

### Experimental animals and sample collection

This study was approved by the Animal Care and Use Committee and the Bioethics Committee of Nippon Veterinary and Life Science University (accession #26 K-29, 27 K-10, 28 K-4; representative researcher was D.H.). FSECs and control cats were housed in the facility for rearing animal at Nippon Veterinary and Life Science University. Founder cats for the FSEC colony were originally purchased from a commercial laboratory (Narc, Inc., Chiba, Japan; already closed down.) in 2009 and have since been maintained as a colony at Nippon Veterinary and Life Science University [7]. All control cats were obtained from two other commercial laboratories (Shiraishi Laboratory Animals Co., Ltd., Saitama, Japan; already closed down, and Liberty Research, Inc., Waverly, NY, USA). For cDNA cloning, brain tissues were obtained from a normal cat unrelated to the FSEC strain and euthanized previously for another study [29]. After euthanasia, brain tissues (cerebral cortex) were collected within 30 min and snap frozen in liquid nitrogen. For mutation analysis, eight FSECs (male:female = 6:2; median age: 95.5 months [range: 82–180 months]; median body weight: 3.9 kg [range: 3.0–5.6 kg]) and eight healthy cats without history of seizures, abnormal findings on magnetic resonance imaging or scalp electroencephalography, or relationship with FSECs (male:female = 5:3; median age: 73 months [range: 33–74 months]; median body weight: 3.8 kg [range: 3.0–6.0 kg]) were included.

A 0.5 ml peripheral blood sample was collected by jugular or inner thigh venipuncture without sedation to extract genomic DNA. The median age of the seizure onset in FSECs was 8 months (range: 3–35 months). Six out of eight FSECs showed both seizure forms, one cat showed only spontaneous limbic seizures evolving into generalized epileptic seizures, and the other showed only vestibular stimulation-induced generalized epileptic seizures. All FSECs used in this study had interictal spikes on scalp electroencephalography, which predominantly arose from the temporal region. All cats were monitored by a 24-h video monitoring system for more than 1 year. Spontaneous seizure frequencies were variable among FSECs (range: 0–36/year). Clinical data of FSECs included in this study are summarized in Table 4.

**Table 4:** Clinical summary of FSECs included in this study

FSECs	Age (months)	Sex	Seizure phenotype	Seizure dominant form	Spontaneous seizure frequency (/year)	Age at Onset (months)	Father	Mother
#1	84	M	SS	SS	About 36	8	Asymptomatic*	Asymptomatic
#2	74	M	SS&VSS	SS	About 24	8	Asymptomatic†	Asymptomatic
#3	61	F	SS&VSS	SS	0–2	10	Asymptomatic†	Asymptomatic
#4	67	M	SS&VSS	VSS	About 2	17	Symptomatic (#1)	Symptomatic (#6)
#5	129	M	SS&VSS	SS	0–4	36	Asymptomatic	Asymptomatic
#6	87	F	SS&VSS	VSS	0–2	8	Asymptomatic*	Asymptomatic
#7	74	M	SS&VSS	VSS	0–1	35	Symptomatic¶	Symptomatic‡¶
#8	79	M	VSS	VSS	N/A	4	Asymptomatic	Symptomatic‡¶

^*, †, ‡^Cats with the same symbol are the same individual. ^¶^Cats showed spontaneous seizures (SS) only. Vestibular-stimulated seizures (VSS) were rare in all cats used in this study. *M* Male. *F* Female

### RNA extraction and first strand cDNA synthesis

Total RNA was extracted from a normal cat brain using Illustra RNA spin columns (GE Healthcare UK Ltd., Little Chalfont, England), according to the manufacturer’s instructions. Total RNA was reverse transcribed into first-strand cDNA using ReverTra Ace reverse transcriptase (Toyobo, Osaka, Japan). The cDNA was used as the template to amplify the fLGI1, 2, 3, and 4 genes.

### Cloning of full-length fLGI cDNAs

To amplify overlapping fragments including ORFs of the fLGI1–4 genes, oligonucleotide primers were designed based on the predicted mRNA sequences of the *Felis catus* LGI1–4 genes (GenBank accession numbers: XM_003994222.3, XM_011281979.1, XM_003984713.3, and XM_003997904.3, respectively) using Primer3.0 online software (http://www.bioinformatics.nl/cgi-bin/primer3plus/primer3plus.cgi/). PCR was performed with AmpliTaq Gold 360 Master Mix (Thermo Fisher Scientific, Waltham, MA, USA) using brain cDNA as a template. PCR cycle conditions were follows: 95 °C for 10 min, followed by 38 cycles of denaturation (95 °C for 30 s), annealing (30 s), and extension (72 °C for 60 s), followed by a final extension (72 °C for 7 min). The set of primers used and each annealing temperature are summarized in Additional file 12. PCR products were detected by 2% agarose gel electrophoresis, isolated from the agarose, and purified using the Wizard SV Gel and PCR Clean-Up System (Promega, Fitchburg, WI, USA).

For TA cloning, purified cDNA were ligated with T4 DNA ligase and cloned into the pGEM-T Easy Vector System (Promega). The vector containing purified PCR product was transformed into competent high DH5α cells (Toyobo). Plasmids were isolated from DH5α cells using the NucleoSpin Plasmid QuickPure Kit (Takara Bio, Shiga, Japan). As the 5′-region of the coding sequence of fLGI2 was not represented in the predicted sequences (GenBank accession numbers: XM_011281979.1), 3′- and 5′-rapid amplification of cDNA ends PCR was performed. The sequences at the 5′-end of LGI2 were amplified using the SMARTer RACE 5′/3’ Kit (Takara Bio), following the manufacturer’s instructions.

### DNA extraction from peripheral blood, PCR for mutation detection, and product purification

Genomic DNA from eight FSECs and eight control cats was extracted from peripheral blood collected in tubes containing ethylenediaminetetraacetic acid using the QIAamp DNA Mini Kit (Qiagen, Hilden, Germany), according to the manufacturer’s instructions. PCR was performed as described above for cloning. Primers (flanking the intron/exon boundaries) were designed based on the predicted genomic sequences for each of the fLGI1–4 genes (NC_018733.2, NC_018726.2, NC_018726.2, and NC_018737.2, respectively) using Primer 3.0 online software. Detection and purification of PCR products was performed as described above for LGI gene cloning, except that the concentration of agarose gel used was 1.8%. Primer sequences and annealing temperatures are shown in Additional file 13.

### Sequencing

Purified DNA and purified PCR products were sequenced using an Applied Biosystems 3130xl Genetic Analyzer (Applied Biosystems, Foster City, CA, USA) or 3730xl DNA Analyzer (Applied Biosystems) using BigDye Terminator v3.1 (Applied Biosystems). Sequencing results were analyzed using the CLC sequence viewer (Version 7.0; CLC Bio, Waltham, MA, USA). For mutation analysis, direct sequencing of the PCR products was performed twice.

### Bioinformatics analyses of fLGI genes

The full-length cDNA sequences of the fLGI genes were assembled using CLC Sequence Viewer. The basic bioinformatics analysis software used in this study are shown in Table 5. The amino acid sequences were deduced from our cDNA sequences. The genomic sequences of LGI1–4 genes from other species (accession numbers shown in Additional file 5) were used to perform exonic sequence structure analysis. All amino acid sequences used for multiple sequence alignment and homology analysis are summarized in Additional file 10. Pairwise alignments between the *Felis catus* and other species were performed with LALIGN, using the following parameters: scoring matrix (BLOSUM50), gap open/extension penalty (−12/−2). Multiple sequence alignments were constructed using DNAMAN version 9.122 (Trial version; Lynnon Biosoft, San Ramon, CA, USA). A phylogenetic analysis was performed using the neighbor-joining methods (bootstrap phylogeny test, 1000 replicates) with MEGA 7.0 software to calculate the p-distance. In silico functional analysis of non-synonymous mutations was performed using SIFT, PP2 and PROVEAN. In SIFT analysis, the amino acid substitution are predicted damaging if the score is ≤0.05, and tolerated if the score is >0.05. PP2 score ranges from 0.0 (tolerated) to 1.0 (deleterious). PROVEAN was used with a threshold value of −2.5.

**Table 5:** List of bioinformatics analysis software used in this study

Bioinformatics analysis software	URL	Prediction purpose
ORF finder	https://www.ncbi.nlm.nih.gov/orffinder/	Predict ORF
ProtParam	http://web.expasy.org/protparam/	Basic properties
PsiPred	http://bioinf.cs.ucl.ac.uk/psipred/	Secondary structures
NetOGlyc 4.0	http://www.cbs.dtu.dk/services/NetOGlyc/	O-glycosylation sites
NetNGlyc 1.0	http://www.cbs.dtu.dk/services/NetNGlyc/	N-glycosylation sites
NetPhos 3.1	http://www.cbs.dtu.dk/services/NetPhos/	Phosphorylation sites
TMHMM Server v. 2.0	http://www.cbs.dtu.dk/services/TMHMM/	Transmembrane domains
SignalP 4.1	http://www.cbs.dtu.dk/services/SignalP/	Signal peptides
SCRATCH	http://scratch.proteomics.ics.uci.edu/	Disulfide bonds
PSORT II	https://psort.hgc.jp/form2.html	Sub cellular distribution
LALIGN	http://www.ch.embnet.org/software/LALIGN_form.html	Pairwise alignments
SMART	http://smart.embl.de/	Structural domains
STRING 10.0	http://string-db.org/	Proteins interaction
SIFT	http://sift.bii.a-star.edu.sg/	Predicting pathogenicity
PP2	http://genetics.bwh.harvard.edu/pph2/	Predicting pathogenicity
PROVEAN	http://provean.jcvi.org	Predicting pathogenicity

## Additional files

Additional file 1: The subcellular distribution of feline leucine-rich glioma-inactivated (fLGI) proteins. The subcellular distribution of fLGI proteins are represented in pie charts. (PDF 301 kb)

Additional file 2: Confidently predicted domains, repeats, and motifs of LGI proteins by SMART (Simple Modular Architecture Research Tool) (DOCX 41 kb)

Additional file 3: The confidence views of the LGI protein interaction. Stronger associations are represented by thicker lines. (**a**) Confidence views of fLGI1 protein interactions, (**b**) fLGI2 protein interactions, (**c**) fLGI3 protein interactions, and (**d**) fLGI4 protein interactions (TIFF 674 kb)

Additional file 4: Exonic structures of the coding sequences of the LGI family genes from *Felis catus* and other species. (**a**–**d**) show comparison of exonic structures between species for the LGI1–LGI4 genes, respectively. Amino acid sequences and genomic sequences are provided in Additional file 5. Colored blocks represent exons. Gray blocks represent introns. Numbers over the colored blocks indicate base pairs that construct exons (TIFF 15627 kb)

Additional file 5 Accession numbers of the amino acid sequences and genomic sequences used for exonic structural analysis (DOCX 84 kb)

Additional file 6 Multiple alignment of LGI1 amino acid sequences from *Felis catus* and other species. Multiple alignment was constructed using DNAMAN 9.122. The black, red, and light blue shading represent 100% conserved, less-conserved (≥75%), and non-conserved (≥50%) amino acids, respectively (JPEG 14409 kb)

Additional file 7 Multiple alignment of LGI2 amino acid sequences from *Felis catus* and other species. Multiple alignment was constructed using DNAMAN 9.122. The black, red, and light blue shading represent 100% conserved, less-conserved (≥75%), and non-conserved (≥50%) amino acids, respectively (JPEG 14074 kb)

Additional file 8 Multiple alignment of LGI3 amino acid sequences from *Felis catus* and other species. Multiple alignment was constructed using DNAMAN 9.122. The black, red, and light blue shading represent 100% conserved, less-conserved (≥75%), and non-conserved (≥50%) amino acids, respectively (JPEG 13184 kb)

Additional file 9 Multiple alignment of LGI4 amino acid sequences from *Felis catus* and other species. Multiple alignment was constructed using DNAMAN 9.122. The black, red, and light blue shading represent 100% conserved, less-conserved (≥75%), and non-conserved (≥50%) amino acids, respectively (JPEG 11862 kb)

Additional file 10 List of amino acid sequences of LGI proteins used in the analysis (DOCX 59 kb)

Additional file 11 Allelic and genotypic distribution of synonymous and intronic polymorphisms other than non-synonymous mutation found in LGI1–4 genes in familial spontaneous epileptic cats (FPSCs) and controls (DOCX 86 kb)

Additional file 12 List of oligonucleotide primers used for molecular cloning of ORFs of fLGI genes. In rapid-amplification of cDNA ends (RACE), touchdown PCR was performed, with annealing temperatures of 70 °C for five cycles and 68 °C for 20 cycles (DOCX 85 kb)

Additional file 13 List of oligonucleotide primers used in sequencing and mutation analysis of the fLGI1–4 genes (DOCX 108 kb)

## Abbreviations

ADAM: A disintegrin and metalloprotease
ADLTE: Autosomal dominant lateral temporal lobe epilepsy
Bp: Base pair(s)
cDNA: DNA complementary to RNA
EPTP: Epitempin
FEPSO: Feline complex partial seizure with orofacial involvement
fLGI: Feline leucine-rich glioma inactivated
FMTLE: Familial mesial temporal lobe epilepsy
FSEC: Familial spontaneous epileptic cats
LE: Limbic encephalitis
LGI: Leucine-rich glioma inactivated
LRR: Leucine rich repeat
ORF: Open reading frame
RACE: Rapid-amplification of cDNA ends
RT-PCR: Reverse transcription-polymerase chain reaction
SNPs: Single nucleotide polymorphisms
TLE: Temporal lobe epilepsy
VGKC: Voltage-gated K Channel
