# Supplementary figures and images for: Molecular cloning and characterization of the family of feline leucine-rich glioma-inactivated (LGI) genes, and mutational analysis in familial spontaneous epileptic cats
Source: BMC Vet Res. 2017 Dec 13;13:389. doi: 10.1186/s12917-017-1308-9 (PMC5729232; doi:10.1186/s12917-017-1308-9)

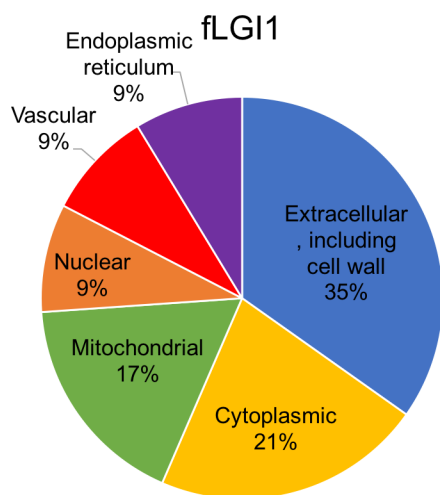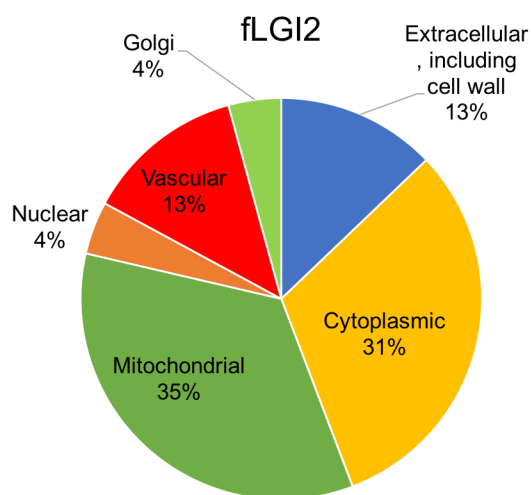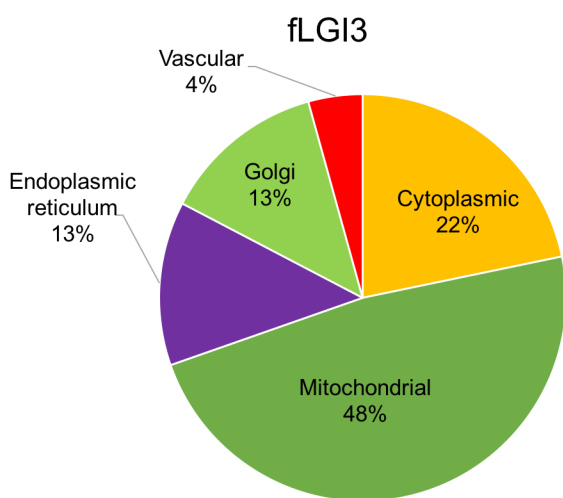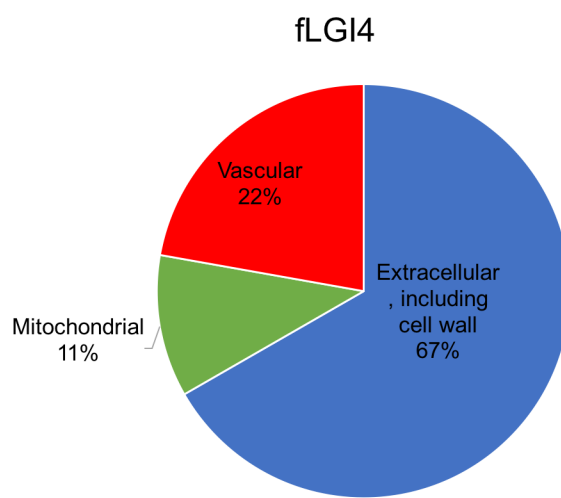

Supplement: Supplementary file 1 — The subcellular distribution of feline leucine-rich glioma-inactivated (fLGI) proteins. The subcellular distribution of fLGI proteins are represented in pie charts. (PDF 301 kb) [file 12917_2017_1308_MOESM1_ESM.pdf]

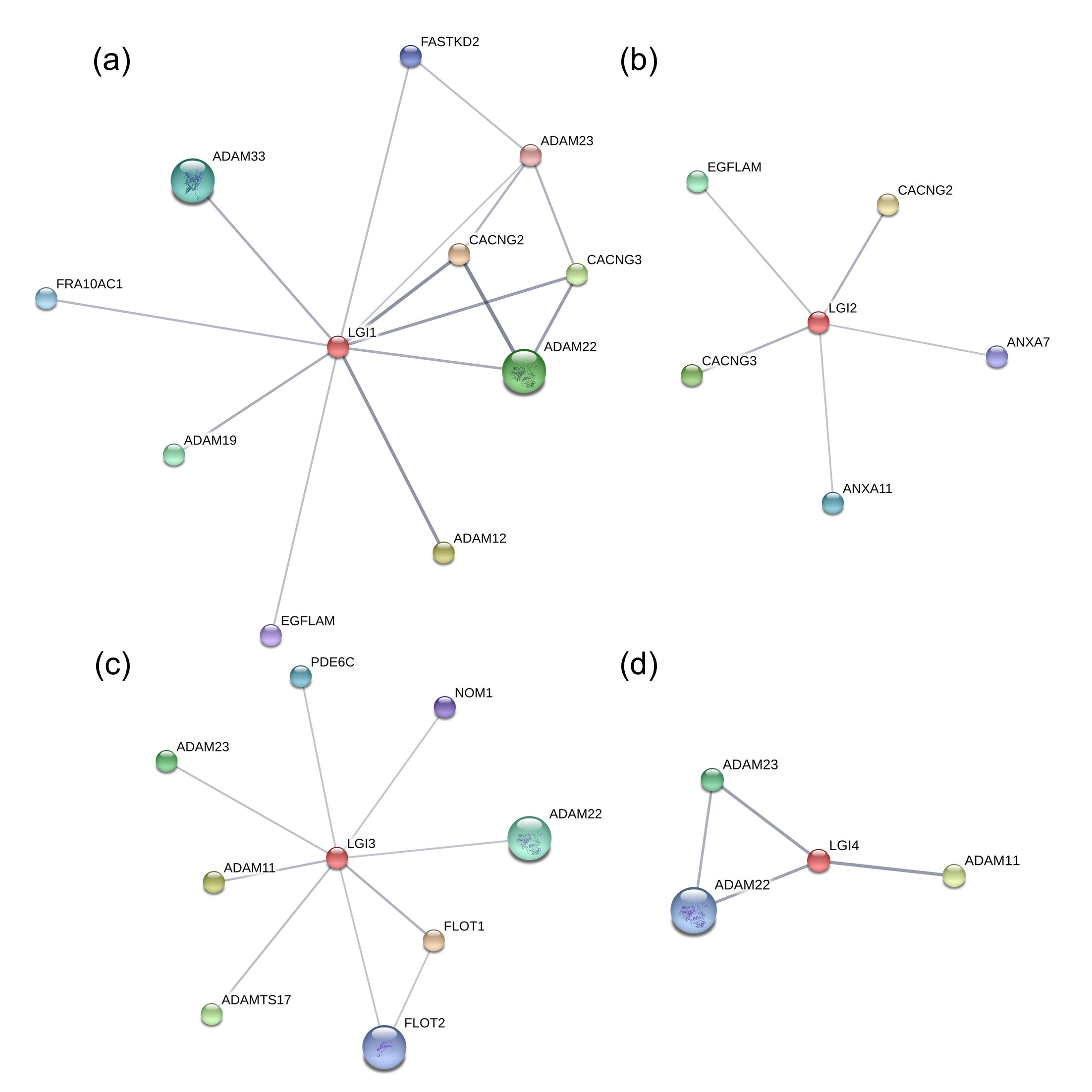

Supplement: Supplementary file 3 — The confidence views of the LGI protein interaction. Stronger associations are represented by thicker lines. (a) Confidence views of fLGI1 protein interactions, (b) fLGI2 protein interactions, (c) fLGI3 protein interactions, and (d) fLGI4 protein interactions (TIFF 674 kb) [file 12917_2017_1308_MOESM3_ESM.tiff]

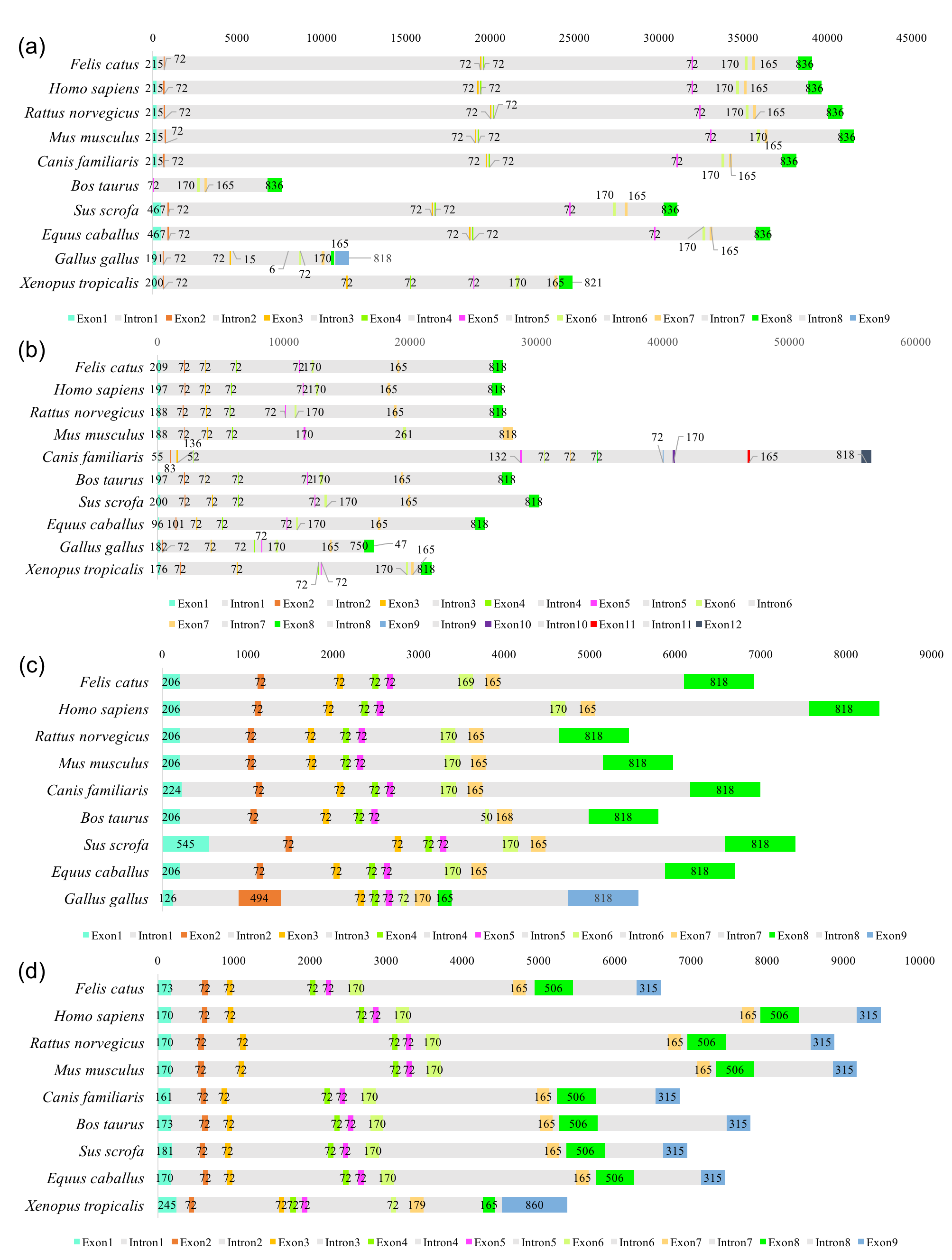

Supplement: Supplementary file 4 — Exonic structures of the coding sequences of the LGI family genes from Felis catus and other species. (a–d) show comparison of exonic structures between species for the LGI1–LGI4 genes, respectively. Amino acid sequences and genomic sequences are provided in Additional file 5. Colored blocks represent exons. Gray blocks represent introns. Numbers over the colored blocks indicate base pairs that construct exons (TIFF 15627 kb) [file 12917_2017_1308_MOESM4_ESM.tiff]
