# Supplementary material for: Molecular cloning and characterization of the family of feline leucine-rich glioma-inactivated (LGI) genes, and mutational analysis in familial spontaneous epileptic cats
Source: BMC Vet Res. 2017 Dec 13;13:389. doi: 10.1186/s12917-017-1308-9 (PMC5729232; doi:10.1186/s12917-017-1308-9)
Supplement: Supplementary file 2 — Confidently predicted domains, repeats, and motifs of LGI proteins by SMART (Simple Modular Architecture Research Tool) (DOCX 41 kb) [file 12917_2017_1308_MOESM2_ESM.docx]

**Additional file 2**

|  | LGI1 | LGI2 | LGI3 | LGI4 |
| --- | --- | --- | --- | --- |
| Leucine rich repeat N-terminal domain | 41–71 | 39–69 |  |  |
| Leucine rich repeat C-terminal domain | 173–222 | 171–220 | 170–219 | 159–208 |
| Leucine-rich repeat, typical subtype | 114–137, 138–161 | 88–111, 112–135, 136–159 | 135–158 | 124–147 |
| Leucine-rich repeats | 90–113 |  | 87–110, 111–134 | 76–99, 100–123 |
| EPTP domain | 225–266, 271–312, 317–363, 366–414, 419–461, 464–505, 510–550 | 223–264, 269–310, 315–361, 364–406, 411–453, 456–497 | 222–263, 268–309, 314–360, 363–405, 410–452, 455–496 | 397–439 |
