# Supplementary material for: Molecular cloning and characterization of the family of feline leucine-rich glioma-inactivated (LGI) genes, and mutational analysis in familial spontaneous epileptic cats
Source: BMC Vet Res. 2017 Dec 13;13:389. doi: 10.1186/s12917-017-1308-9 (PMC5729232; doi:10.1186/s12917-017-1308-9)
Supplement: Supplementary file 5 — Accession numbers of the amino acid sequences and genomic sequences used for exonic structural analysis (DOCX 84 kb) [file 12917_2017_1308_MOESM5_ESM.docx]

| Gene | Species | Amino acid sequence number | Chromosome name | Accession numbers |
| --- | --- | --- | --- | --- |
| *LGI1* | *Felis catus* |  | *F. Catus* chromosome D2, Felis_catus_8.0 | NC_018733.2 |
|  | *Homo sapiens* | NP_005088.1 | *H. sapiens* chromosome 10, GRCh38.p7 | NC_000010.11 |
|  | *Rattus norvegicus* | NP_665712.1 | *R. norvegicus* chromosome 1, Rnor_6.0 | NC_005100.4 |
|  | *Mus musculus* | NP_064674.1 | *M. musculus* strain C57BL/6J chromosome 19, GRCm38.p4 C57BL/6J | NC_000085.6 |
|  | *Canis familiaris* | XP_534971.2 | *Canis lupus familiaris* chromosome 28, CanFam3.1 | NC_006610.3 |
|  | *Bos taurus* | NP_001040056.2 | *B. taurus* chromosome 26, Bos_raurus_UMD_3.1.1 | AC_000183.1 |
|  | *Sus scrofa* | XP_001928756.1 | *S. Scrofa* chromosome 14, Sscrofa 11.1 | NC_010456.4 |
|  | *Equus caballus* | XP_001502450.3 | *E. caballus* chromosome 1, EquCab2.0 | NC_009144.2 |
|  | *Gallus gallus* | NP_001038120.1 | *G. gallus* chromosome 6, Gallus_gallus-5.0 | NC_006093.4 |
|  | *Xenopus tropicalis* | NP_001072366.1 | *X. tropicalis* chromosome 7, Xenopus_tropicalis_v9.1 | NC_030683.1 |
| *LGI2* | *Felis catus* |  | *F. Catus* chromosome B1, Felis_catus_8.0 | NC_018726.2 |
|  | *Homo sapiens* | NP_060646.2 | *H. sapiens* chromosome 4, GRCh38.p7 | NC_000004.12 |
|  | *Rattus norvegicus* | NP_001100689.2 | *R. norvegicus* chromosome 14, Rnor_6.0 | NC_005113.4 |
|  | *Mus musculus* | NP_659194.1 | *M. musculus* strain C57BL/6J chromosome 5, GRCm38.p4 C57BL/6J | NC_000071.6 |
|  | *Canis familiaris* | XP_013967830.1 | *Canis lupus familiaris* chromosome 3, CanFam3.1 | NC_006585.3 |
|  | *Bos taurus* | NP_001179745.1 | *B. taurus* chromosome 6, Bos_raurus_UMD_3.1.1 | AC_000163.1 |
|  | *Sus scrofa* | XP_020956221.1 | *S. Scrofa* chromosome 8, Sscrofa 11.1 | NC_010450.4 |
|  | *Equus caballus* | XP_014594043.1 | *E. caballus* chromosome 3, EquCab2.0 | NC_009146.2 |
|  | *Gallus gallus* | NP_001244245.1 | *G. gallus* chromosome 4, Gallus_gallus-5.0 | NC_006091.4 |
|  | *Xenopus tropicalis* | NP_001096394.1 | *X. tropicalis* chromosome 1, Xenopus_tropicalis_v9.1 | NC_030677.1 |
| *LGI3* | *Felis catus* |  | *F. Catus* chromosome B1, Felis_catus_8.0 | NC_018726.2 |
|  | *Homo sapiens* | NP_644807.1 | *H. sapiens* chromosome 8, GRCh38.p7 | NC_000008.11 |
|  | *Rattus norvegicus* | NP_001100747.1 | *R. norvegicus* chromosome 15, Rnor_6.0 | NC_005114.4 |
|  | *Mus musculus* | NP_660254.1 | *M. musculus* strain C57BL/6J chromosome 14, GRCm38.p4 C57BL/6J | NC_000080.6 |
|  | *Canis familiaris* | XP_543254.3 | *Canis lupus familiaris* chromosome 25, CanFam3.1 | NC_006607.3 |
|  | *Bos taurus* | XP_015320076.1 | *B. taurus* chromosome 8, Bos_raurus_UMD_3.1.1 | AC_000165.1 |
|  | *Sus scrofa* | XP_003132845.2 | *S. Scrofa* chromosome 14, Sscrofa 11.1 | NC_010456.5 |
|  | *Equus caballus* | XP_001491042.1 | *E. caballus* chromosome 2, EquCab2.0 | NC_009145.2 |
|  | *Gallus gallus* | XP_015128838.1 | *G. gallus* Unplaced Scaffold, Gallus_gallus-5.0 | NT_464503.1 |
| *LGI4* | *Felis catus* |  | *F. Catus* chromosome E2, Felis_catus_8.0 | NC_018737.2 |
|  | *Homo sapiens* | NP_644813.1 | *H. sapiens* chromosome 19, GRCh38.p7 | NC_000019.10 |
|  | *Rattus norvegicus* | NP_955793.1 | *R. norvegicus* chromosome 1, Rnor_6.0 | NC_005100.4 |
|  | *Mus musculus* | NP_653139.2 | *M. musculus* strain C57BL/6J chromosome 7, GRCm38.p4 C57BL/6J | NC_000073.6 |
|  | *Canis familiaris* | XP_541696.2 | *Canis lupus familiaris* chromosome 1, CanFam3.1 | NC_006583.3 |
|  | *Bos taurus* | NP_001096771.1 | *B. taurus* chromosome 18, Bos_raurus_UMD_3.1.1 | AC_000175.1 |
|  | *Sus scrofa* | XP_020952816.1 | *S. Scrofa* chromosome 6, Sscrofa 11.1 | NC_010448.4 |
|  | *Equus caballus* | XP_001491349.1 | *E. caballus* chromosome 10, EquCab2.0 | NC_009153.2 |
|  | *Xenopus tropicalis* | NP_644813.1 | *X. tropicalis* chromosome 7, Xenopus_tropicalis_v9.1 | NC_030683.1 |

**Additional file 5**
