# Supplementary material for: Molecular cloning and characterization of the family of feline leucine-rich glioma-inactivated (LGI) genes, and mutational analysis in familial spontaneous epileptic cats
Source: BMC Vet Res. 2017 Dec 13;13:389. doi: 10.1186/s12917-017-1308-9 (PMC5729232; doi:10.1186/s12917-017-1308-9)
Supplement: Supplementary file 10 — List of amino acid sequences of LGI proteins used in the analysis (DOCX 59 kb) [file 12917_2017_1308_MOESM10_ESM.docx]

**Additional file 10**

| Species | LGI1 | LGI2 | LGI3 | LGI4 |
| --- | --- | --- | --- | --- |
| *Homo sapiens* | NP_005088.1 | NP_060646.2 | NP_644807.1 | NP_644813.1 |
| *Rattus norvegicus* | NP_665712.1 | NP_001100689.2 | NP_001100747.1 | NP_955793.1 |
| *Mus musculus* | NP_064674.1 | NP_659194.1 | NP_660254.1 | NP_653139.2 |
| *Canis familiaris* | XP_534971.2 | XP_013967830.1 | XP_543254.3 | XP_541696.2 |
| *Bos taurus* | NP_001040056.2 | NP_001179745.1 | XP_015320076.1 | NP_001096771.1 |
| *Sus scrofa* | XP_001928756.1 | XP_020956221.1 | XP_003132845.2 | XP_020952816.1 |
| *Equus caballus* | XP_001502450.3 | XP_014594043.1 | XP_001491042.1 | XP_001491349.1 |
| *Gallus gallus* | NP_001038120.1 | NP_001244245.1 | XP_015128838.1 | N/A |
| *Xenopus tropicalis* | NP_001072366.1 | NP_001096394.1 | N/A | NP_001039090.1 |

N/A: Not available
