# Supplementary material for: Molecular cloning and characterization of the family of feline leucine-rich glioma-inactivated (LGI) genes, and mutational analysis in familial spontaneous epileptic cats
Source: BMC Vet Res. 2017 Dec 13;13:389. doi: 10.1186/s12917-017-1308-9 (PMC5729232; doi:10.1186/s12917-017-1308-9)
Supplement: Supplementary file 11 — Allelic and genotypic distribution of synonymous and intronic polymorphisms other than non-synonymous mutation found in LGI1–4 genes in familial spontaneous epileptic cats (FPSCs) and controls (DOCX 86 kb) [file 12917_2017_1308_MOESM11_ESM.docx]

**Additional File 11**

|  | Nucleotide | Amino Acid | Accession # | Group (n) | Genotype frequencies | | | Allele frequencies | |
| --- | --- | --- | --- | --- | --- | --- | --- | --- | --- |
| LGI1 |  |  |  |  | G/G | G/T | T/T | f(G) | f(T) |
|  | c.431+9G>T | — | — | FSECs(8) | 8 | 0 | 0 | 1 | 0 |
|  |  |  |  | Controls(8) | 6 | 2 | 0 | 0.875 | 0.125 |
|  |  |  |  |  | TTTG |  | del |  |  |
|  | c.431+51_55del | — | — | FSECs(8) | 8 |  | 0 | — | — |
|  |  |  |  | Controls(8) | 7 |  | 1 | — | — |
|  |  |  |  |  | T/T | T/C | C/C | f(T) | f(C) |
|  | c.492T>C | p.S164S | — | FSECs(8) | 4 | 3 | 1 | 0.6875 | 0.3125 |
|  |  |  |  | Controls(8) | 8 | 0 | 0 | 1 | 0 |
| LGI2 |  |  |  |  | T/T | T/G | G/G | f(T) | f(G) |
|  | c.281+20T>G | — | — | FSECs(8) | 8 | 0 | 0 | 1 | 0 |
|  |  |  |  | Controls(8) | 7 | 1 | 0 | 0.9375 | 0.0625 |
|  |  |  |  |  | T/T | T/G | G/G | f(T) | f(G) |
|  | c.281+67T>G | — | — | FSECs(8) | 6 | 2 | 0 | 0.875 | 0.125 |
|  |  |  |  | Controls(8) | 4 | 4 | 0 | 0.5 | 0.5 |
|  |  |  |  |  | T/T | T/C | C/C | f(T) | f(C) |
|  | c.353+31T>C | — | — | FSECs(8) | 0 | 2 | 6 | 0.125 | 0.875 |
|  |  |  |  | Controls(8) | 0 | 3 | 5 | 0.1875 | 0.8125 |
|  |  |  |  |  | T/T | T/C | C/C | f(T) | f(C) |
|  | c.497+15T>C | — | — | FSECs(8) | 8 | 0 | 0 | 1 | 0 |
|  |  |  |  | Controls(8) | 7 | 1 | 0 | 0.9375 | 0.0625 |
|  |  |  |  |  | A/A | A/G | G/G | f(A) | f(G) |
|  | c.497+21A>G | — | — | FSECs(8) | 7 | 1 | 0 | 0.9375 | 0.0625 |
|  |  |  |  | Controls(8) | 5 | 3 | 0 | 0.8125 | 0.1875 |
|  |  |  |  |  | C/C | C/T | T/T | f(C) | f(T) |
|  | c.501C>T | p.D167D | — | FSECs(8) | 8 | 0 | 0 | 1 | 0 |
|  |  |  |  | Controls(8) | 6 | 1 | 1 | 0.8125 | 0.1875 |
|  |  |  |  |  | G/G | G/T | T/T | f(G) | f(T) |
|  | c.576G>T | p.T192T | — | FSECs(8) | 4 | 4 | 0 | 0.75 | 0.25 |
|  |  |  |  | Controls(8) | 4 | 4 | 0 | 0.75 | 0.25 |
|  |  |  |  |  | G/G | G/T | T/T | f(G) | f(T) |
|  | c.667+19G>T | — | — | FSECs(8) | 8 | 0 | 0 | 1 | 0 |
|  |  |  |  | Controls(8) | 7 | 1 | 0 | 0.9375 | 0.0625 |
|  |  |  |  |  | G/G | G/A | A/A | f(G) | f(A) |
|  | c.813G>A | p.R271R | — | FSECs(8) | 8 | 0 | 0 | 1 | 0 |
|  |  |  |  | Controls(8) | 6 | 1 | 1 | 0.8125 | 0.1875 |
|  |  |  |  |  | T/T | T/C | C/C | f(T) | f(C) |
|  | c.813+22T>C | — | — | FSECs(8) | 8 | 0 | 0 | 1 | 0 |
|  |  |  |  | Controls(8) | 6 | 1 | 1 | 0.8125 | 0.1875 |
|  |  |  |  |  | C/C | C/T | T/T | f(C) | f(T) |
|  | c.906C>T | p.G302G | — | FSECs(8) | 8 | 0 | 0 | 1 | 0 |
|  |  |  |  | Controls(8) | 7 | 1 | 0 | 0.9375 | 0.0625 |
|  |  |  |  |  | T/T | T/C | C/C | f(T) | f(C) |
|  | c.1317T>C | p.S439S | — | FSECs(8) | 7 | 1 | 0 | 0.9375 | 0.0625 |
|  |  |  |  | Controls(8) | 6 | 2 | 0 | 0.875 | 0.125 |
|  |  |  |  |  | C/C | C/G | G/G | f(C) | f(G) |
|  | c.1398C>G | p.R466R | — | FSECs(8) | 8 | 0 | 0 | 1 | 0 |
|  |  |  |  | Controls(8) | 4 | 0 | 4 | 0.5 | 0.5 |
|  |  |  |  |  | A/A | A/G | G/G | f(A) | f(G) |
|  | c.*32A>G | — | — | FSECs(8) | 2 | 2 | 4 | 0.375 | 0.625 |
|  |  |  |  | Controls(8) | 0 | 4 | 4 | 0.5 | 0.5 |
|  |  |  |  |  | C/C | C/T | T/T | f(C) | f(T) |
|  | c.*53C>T | — | — | FSECs(8) | 8 | 0 | 0 | 1 | 0 |
|  |  |  |  | Controls(8) | 7 | 1 | 0 | 0.9375 | 0.0625 |
|  |  |  |  |  | G/G | G/A | A/A | f(G) | f(A) |
|  | c.*55G>A | — | — | FSECs(8) | 4 | 4 | 0 | 0.5 | 0.5 |
|  |  |  |  | Controls(8) | 2 | 3 | 3 | 0.4375 | 0.5625 |
|  |  |  |  |  | C/C | C/A | A/A | f(C) | f(A) |
|  | c.*57G>A | — | — | FSECs(8) | 8 | 0 | 0 | 1 | 0 |
|  |  |  |  | Controls(8) | 7 | 1 | 0 | 0.9375 | 0.0625 |
| LGI3 |  |  |  |  | G/G | G/A | A/A | f(G) | f(A) |
|  | c.-87G>A | — | — | FSECs(8) | 8 | 0 | 0 | 1 | 0 |
|  |  |  |  | Controls(8) | 5 | 3 | 0 | 0.8125 | 0.1875 |
|  |  |  |  |  | C/C | C/G | G/G | f(C) | f(G) |
|  | c.350+134C>G | — | — | FSECs(8) | 8 | 0 | 0 | 1 | 0 |
|  |  |  |  | Controls(8) | 5 | 3 | 0 | 0.8125 | 0.1875 |
|  |  |  |  |  | T/T | T/C | C/C | f(T) | f(C) |
|  | c.422+27T>C | — | — | FSECs(8) | 4 | 1 | 3 | 0.5625 | 0.4375 |
|  |  |  |  | Controls(8) | 6 | 1 | 1 | 0.8125 | 0.1875 |
|  |  |  |  |  | A/A | A/G | G/G | f(A) | f(G) |
|  | c.494+44A>G | — | — | FSECs(8) | 4 | 1 | 3 | 0.5625 | 0.4375 |
|  |  |  |  | Controls(8) | 6 | 1 | 1 | 0.8125 | 0.1875 |
|  |  |  |  |  | G/G | G/A | A/A | f(G) | f(A) |
|  | c.600G>A | p.P200P | — | FSECs(8) | 4 | 1 | 3 | 0.5625 | 0.4375 |
|  |  |  |  | Controls(8) | 5 | 2 | 1 | 0.75 | 0.25 |
|  |  |  |  |  | C/C | C/T | T/T | f(C) | f(T) |
|  | c.1017C>T | p.G339G | — | FSECs(8) | 8 | 0 | 0 | 1 | 0 |
|  |  |  |  | Controls(8) | 7 | 1 | 0 | 0.9375 | 0.0625 |
|  |  |  |  |  | T/T | T/G | G/G | f(T) | f(G) |
|  | c.1056T>G | p.G352G | — | FSECs(8) | 4 | 2 | 2 | 0.625 | 0.375 |
|  |  |  |  | Controls(8) | 2 | 2 | 4 | 0.375 | 0.625 |
|  |  |  |  |  | T/T | T/C | C/C | f(T) | f(C) |
|  | c.1582T>C | p.L528L | rs785566903 | FSECs(8) | 2 | 2 | 4 | 0.375 | 0.625 |
|  |  |  |  | Controls(8) | 2 | 4 | 2 | 0.5 | 0.5 |
| LGI4 |  |  |  |  | C/C | C/T | T/T | f(C) | f(T) |
|  | c.174-9C>T | — | — | FSECs(8) | 4 | 4 | 0 | 0.5 | 0.5 |
|  |  |  |  | Controls(8) | 7 | 1 | 0 | 0.9375 | 0.0625 |
|  |  |  |  |  | A/A | A/G | G/G | f(A) | f(G) |
|  | c.318-46A>G | — | — | FSECs(8) | 4 | 4 | 0 | 0.5 | 0.5 |
|  |  |  |  | Controls(8) | 7 | 0 | 1 | 0.875 | 0.125 |
|  |  |  |  |  | T/T | T/C | C/C | f(T) | f(C) |
|  | c.318-9T>C | — | rs785273308 | FSECs(8) | 4 | 4 | 0 | 0.5 | 0.5 |
|  |  |  |  | Controls(8) | 5 | 2 | 1 | 0.75 | 0.25 |
|  |  |  |  |  | A/A | A/G | G/G | f(A) | f(G) |
|  | c.389+61A>G | — | — | FSECs(8) | 4 | 4 | 0 | 0.5 | 0.5 |
|  |  |  |  | Controls(8) | 7 | 1 | 0 | 0.9375 | 0.0625 |
|  |  |  |  |  | A/A | A/C | C/C | f(A) | f(C) |
|  | c.390-55A>C | — | rs785205043 | FSECs(8) | 4 | 4 | 0 | 0.5 | 0.5 |
|  |  |  |  | Controls(8) | 5 | 2 | 1 | 0.75 | 0.25 |
|  |  |  |  |  | G/G | G/A | A/A | f(G) | f(A) |
|  | c.513G>A | p.L171L | — | FSECs(8) | 4 | 4 | 0 | 0.5 | 0.5 |
|  |  |  |  | Controls(8) | 7 | 1 | 0 | 0.9375 | 0.0625 |
|  |  |  |  |  | A/A | A/G | G/G | f(A) | f(G) |
|  | c.631+62A>G | — | — | FSECs(8) | 4 | 4 | 0 | 0.5 | 0.5 |
|  |  |  |  | Controls(8) | 7 | 1 | 0 | 0.9375 | 0.0625 |
|  |  |  |  |  | A/A | A/G | G/G | f(A) | f(G) |
|  | c.632-68A>G | — | — | FSECs(8) | 4 | 4 | 0 | 0.5 | 0.5 |
|  |  |  |  | Controls(8) | 7 | 1 | 0 | 0.9375 | 0.0625 |
|  |  |  |  |  | C/C | C/T | T/T | f(C) | f(T) |
|  | c.1008C>T | p.D336D | — | FSECs(8) | 7 | 1 | 0 | 0.9375 | 0.0625 |
|  |  |  |  | Controls(8) | 4 | 4 | 0 | 0.5 | 0.5 |
|  |  |  |  |  | G/G | G/A | A/A | f(G) | f(A) |
|  | c.1137G>A | p.L378L | — | FSECs(8) | 6 | 2 | 0 | 0.875 | 0.125 |
|  |  |  |  | Controls(8) | 0 | 6 | 2 | 0.375 | 0.625 |
