# Supplementary material for: Molecular cloning and characterization of the family of feline leucine-rich glioma-inactivated (LGI) genes, and mutational analysis in familial spontaneous epileptic cats
Source: BMC Vet Res. 2017 Dec 13;13:389. doi: 10.1186/s12917-017-1308-9 (PMC5729232; doi:10.1186/s12917-017-1308-9)
Supplement: Supplementary file 12 — List of oligonucleotide primers used for molecular cloning of ORFs of fLGI genes. In rapid-amplification of cDNA ends (RACE), touchdown PCR was performed, with annealing temperatures of 70 °C for five cycles and 68 °C for 20 cycles (DOCX 85 kb) [file 12917_2017_1308_MOESM12_ESM.docx]

**Additional file 12**

| Primer | Sequence (5’-3’) | Product size (bp) | Annealing Temperature (°C) | |
| --- | --- | --- | --- | --- |
| LGI1-R1-F | CCAGAAGCCCTGTTCATGTT | 598 | | 53 |
| LGI1-R1-R | TCCACCAGCCACTTCAGTTT |  |  |  |
| LGI1-R2-F | TTTCAAAGGCCTGGATTCTTT | 591 | | 51 |
| LGI1-R2-R | CCAGCTTTTGAACTGTCAGC |  |  |  |
| LGI1-R3-F | CCAGAAGCCCTGTTCATGTT | 594 | | 51 |
| LGI1-R3-R | TCCACCAGCCACTTCAGTTT |  |  |  |
| LGI1-R4-F | AACTGGGATGCAGAAAAAGC | 574 | | 51 |
| LGI1-R4-R | TCAGCATCCATCACCAAGAA |  |  |  |
| LGI2-5’RACE | GATGTCGCCCGGCACGATCCTGGGC | 229 | | * |
| LGI2-R1-F | CACTTGCAGCTGTACCAAGG | 473 | | 57 |
| LGI2-R1-R | CGATGCACAGGACGTCAG |  |  |  |
| LGI2-R2-F | CTCCCAAGGGATGTCTTCAG | 592 | | 57 |
| LGI2-R2-R | TGTCTGCGATGACGAAGAAC |  |  |  |
| LGI2-R3-F | CCTCATTGACGACCAGGTTT | 592 | | 57 |
| LGI2-R3-R | CAGGGCCAGGTAGTGGTTAT |  |  |  |
| LGI2-R4-F | TGCAGAATGCCCTCTACCTT | 566 | | 57 |
| LGI2-R4-R | TAAATGGGAGAACGGACACG |  |  |  |
| LGI3-R1-F | AGAGCTTGCTTGCAGTCAG | 403 | | 51 |
| LGI3-R1-R | GATAGTGCCCAGATGTCATT |  |  |  |
| LGI3-R2-F | GTTGCTCAACTCCAACAAGT | 600 | | 52 |
| LGI3-R2-R | TACAGCTGGCTGTCTACCA |  |  |  |
| LGI3-R3-F | TCCTCAAGTGGGACTATGTT | 568 | | 52 |
| LGI3-R3-R | ATCTTGGAGTCACCGATGTA |  |  |  |
| LGI3-R4-F | CTGTGAAACACTTCCGTGCT | 600 | | 52 |
| LGI3-R4-R | CCTGGTGCGTATACAAGTCT |  |  |  |
| LGI4-R1-F | CTGGCATGGGACGTGTAGTG | 549 | | 58 |
| LGI4-R1-R | GAGGTCCACATGAGTTAGGGTCT |  |  |  |
| LGI4-R2-F | GGACTTCGCTCACTCACACA | 708 | | 55 |
| LGI4-R2-R | GCTCTGGCGAGGGTAGAAG |  |  |  |
| LGI4-R3-F | GTCCTCACCTGGGACTACAG | 696 | | 55 |
| LGI4-R3-R | GACCTGACTGAAGGCGAAGT |  |  |  |
| LGI4-R4-F | GCTACATCGGGGACTCCAT | 491 | | 50 |
| LGI4-R4-R | GTCTTGAACTCGGCCTGTG |  |  |  |
