# Supplementary material for: Molecular cloning and characterization of the family of feline leucine-rich glioma-inactivated (LGI) genes, and mutational analysis in familial spontaneous epileptic cats
Source: BMC Vet Res. 2017 Dec 13;13:389. doi: 10.1186/s12917-017-1308-9 (PMC5729232; doi:10.1186/s12917-017-1308-9)
Supplement: Supplementary file 13 — List of oligonucleotide primers used in sequencing and mutation analysis of the fLGI1–4 genes (DOCX 108 kb) [file 12917_2017_1308_MOESM13_ESM.docx]

**Additional file 13**

| Primer | | Sequence (5’-3’) | Product size (bp) | Annealing Temperature (°C) |
| --- | --- | --- | --- | --- |
| LGI1 |  |  |  |  |
| Exon1 | Forward | AAAGCCATCCTATTTTTGGTAAC | 683 | 55 |
|  | Reverse | CCCTCCTACCGATACATACAAA |  |  |
| Exon2 | Forward | GCTAAACCGGATTAACATAAGG | 287 | 50 |
|  | Reverse | CTTTTTCTTTCCAGAAACACAGT |  |  |
| Exon3&4 | Forward | GACAGACACCTAGACACTTTTCC | 496 | 55 |
|  | Reverse | AAGTATGCATGGAGAGAAATCAT |  |  |
| Exon5 | Forward | TCGGGAAAGAATAAAAGGGACTTAT | 248 | 58 |
|  | Reverse | ATTCTAATCCTCAATACCAGGCTTC |  |  |
| Exon6 | Forward | CATGTGTTAATTGCTGAAGTAAAGT | 372 | 48 |
|  | Reverse | TTTTAAAAATTAAATCGGTCAGTTG |  |  |
| Exon7 | Forward | TTGTGTGTTTAAAAGCAAAGTAAA | 384 | 52 |
|  | Reverse | CCCCTATACCACTTGTCTTTTTC |  |  |
| Exon8_1 | Forward | CTGTTCAACCAAGGAAATCTCTTAT | 667 | 52 |
|  | Reverse | ATTTCATTACTTTGGAATCACCAAT |  |  |
| Exon8_2 | Forward | CAACACAATTATTCACTAACCAAAC | 586 | 52 |
|  | Reverse | CAGTTAAGGATGGAGATACTGGTAG |  |  |
| LGI2 |  |  |  |  |
| Exon2 | Forward | AGTGGAGGAGTTTGAAATACAG | 198 | 54 |
|  | Reverse | AGAGAGAACATCCCATACTCAC |  |  |
| Exon3 | Forward | GTAAATCTCTGCATTGAAATCTGTC | 200 | 56 |
|  | Reverse | CTCTAAGAGATGCTGTTCCTGCT |  |  |
| Exon4 | Forward | GAATGATACTGCTGTTCTCTTTAG | 181 | 54 |
|  | Reverse | CTTAGTTTCCTTGAGAGAATGTTAC |  |  |
| Exon5 | Forward | GCCTGAGGATAATAACCACATTAAC | 290 | 54 |
|  | Reverse | CTCTATGAAGAGTCAAATGCTCAAC |  |  |
| Exon6 | Forward | GATGTTAAATCATCGTATTCCTGCT | 399 | 54 |
|  | Reverse | CTTTAGAACGCTTAAATGTCACCTA |  |  |
| Exon7 | Forward | TGTCTCAGAGAAGAGAATGTAGAAG | 593 | 52 |
|  | Reverse | GTTTCTGTTCTCACCAATAACTGAC |  |  |
| Exon8_1 | Forward | CTTTACATCTCTCCCCGTTTTAC | 498 | 52 |
|  | Reverse | CTTTTATTCCACTGGAGGATGAT |  |  |
| Exon8_2 | Forward | AAAGGATTCTACTCCTACCAGTC | 566 | 52 |
|  | Reverse | CTTCACAAACTTAAGTCAACGACTA |  |  |
| LGI3 |  |  |  |  |
| Exon1 | Forward | GAGGGTTGGAGGACTCG | 480 | 53 |
|  | Reverse | CTCCCCACGCTTTGTAG |  |  |
| Exon2 | Forward | GTTTATGAGGAGAAGAGAAAGATAG | 398 | 52 |
|  | Reverse | CTATCCCATCCAGTAAAAGTAAG |  |  |
| Exon3 | Forward | CTGTTTCTACCCTTGAAGAAGAT | 520 | 52 |
|  | Reverse | ATTCTGATTCTGGAGACATTAAG |  |  |
| Exon4&5 | Forward | CAGAAATCTAGATGGAGCAGAG | 500 | 53 |
|  | Reverse | CATAGTGTCTCGTGTGTATCAAC |  |  |
| Exon6&7 | Forward | ACCTACTTCTAAAAACCTAGAGAGC | 672 | 54 |
|  | Reverse | CTCTCTACCTATAGGAACCCACT |  |  |
| Exon8_1 | Forward | AGACAGATTATTAGCTGTGAAAAGT | 693 | 51 |
|  | Reverse | ATCTTGGAGTCACCGATGTA |  |  |
| Exon8_2 | Forward | ACCTGTCATCTATCAGTGGAG | 680 | 53 |
|  | Reverse | CCTGGTGCGTATACAAGTCT |  |  |
| LGI4 |  |  |  |  |
| Exon1 | Forward | CTGGCATGGGACGTGTAGT | 379 | 53 |
|  | Reverse | ATCCCAACATCCTTTGCTG |  |  |
| Exon2&3 | Forward | CTGGGATGTAAAGGAAATACAT | 615 | 52 |
|  | Reverse | CGTGCCAGTAAGTCTCTATACC |  |  |
| Exon4&5 | Forward | CTCCTGGGATTGTAGTCTGG | 535 | 53 |
|  | Reverse | AACTTAGTTATGTCAACCGAAAGT |  |  |
| Exon6 | Forward | GTCCTCACATATTTCCTCACTC | 498 | 52 |
|  | Reverse | ACTGTTTATTACCTCCATTTTACAG |  |  |
| Exon7&8 | Forward | CTGTAGATCAAAAGATTCGCTAT | 695 | 52 |
|  | Reverse | CTCTGGCGAGGGTAGAAG |  |  |
| Exon8 | Forward | GTGTCCTGCAAGCCACTG | 588 | 55 |
|  | Reverse | GTCGGTGCTGAAAGACCA |  |  |
| Exon9 | Forward | GTGACATGTGTGAAGGTAAGAA | 639 | 52 |
|  | Reverse | GTTGCTTCAGACTGGAAGTT |  |  |
